# Supplementary material for: Magnetically assisted drop-on-demand 3D printing of microstructured multimaterial composites
Source: Nat Commun. 2022 Aug 26;13:5015. doi: 10.1038/s41467-022-32792-1 (PMC9418172; doi:10.1038/s41467-022-32792-1)
Supplement: Supplementary file 1 — Supplementary Information [file 41467_2022_32792_MOESM1_ESM.pdf]

Supplementary Information

Magnetically assisted drop-on-demand 3D printing of microstructured multimaterial composites

Wing Chung Liu[1], Vanessa Hui Yin Chou[1], Rohit Pratyush Behera[1], Hortense Le Ferrand[1,2*]

[1] *School of Mechanical and Aerospace Engineering, Nanyang Technological University, Singapore 639798*

[2] *School of Materials Science and Engineering, Nanyang Technological University of Singapore, Singapore 639798*

*Corresponding authors: hortense@ntu.edu.sg

This supplementary file contains, in order:

- Supplementary Discussion
- Supplementary Figures
- Supplementary Tables
- Supplementary References

Supplementary Discussion

Calculation of the microplatelet volume fraction in drying droplets

The evolution of ϕ during the drying process was estimated by extracting the apparent volume of the droplets during drying. By conservation of mass, we estimate ϕ using:

$$\phi_i V_i = \phi_t V_t \tag{1}$$

where ϕ represents the microplatelet volume fraction, V represents the volume of the droplet. The subscripts i and t refer to the state of the droplet initially upon deposition and at any time t during drying.

Since we control ϕ_i and V_i, we can obtain ϕ_t, by estimating the V_t from the optical micrographs. This volume is outlined by the black dotted lines in the upper panel of Figure 2a. During settling, the volume is approximated as a spherical cap and when the platelets are fully settled, the volume is approximated as a cylinder.

Description of the model for critical magnetic field calculation

This section outlines the calculations done to estimate B_{crit} required for good alignment. The general steps involve estimating an average capillary flow velocity, converting the flow to an equivalent torque on the microplatelet, and then balancing all the torques acting on the microplatelet.

First, the capillary velocity v_{cap}, is estimated using a model developed by Deegan et al.[1] v_{cap} is a function of the time t, distance from the droplet centre r, fluid density ρ, position of the air-liquid interface h, droplet radius R, rate of evaporative mass loss per unit area per unit time J_s and contact angle θ_c:

$$v_{cap}(r,t) = -\frac{1}{\rho r h}\int_0^r r\left(J_s(r)\sqrt{1+\left(\frac{\partial h}{\partial r}\right)^2} + \rho\frac{\partial h}{\partial t}\right) dr \tag{2}$$

$$J_s(r) = J_0\left[1-\left(\frac{r}{R}\right)^2\right]^{-\lambda} \tag{3}$$

$$\lambda = \frac{\pi - 2\theta_c}{2\pi - 2\theta_c} \tag{4}$$

The constant J_0 can be approximated by an analytical solution[2]:

$$J_0 = f(\theta_c)\frac{D(1-H)c_v}{R} \tag{5}$$

where D = diffusivity of water vapour in air, H = relative humidity of surrounding air, c_v = saturation concentration of water vapour in air.

The differentials within the integral equation (2) can be calculated assuming the droplet shape is a spherical cap which is mathematically described by:

$$h(r,t) = \sqrt{\frac{R^2}{sin^2\theta_c} - r^2} - \frac{R}{tan\theta_c} \quad (6)$$

The calculated v_{cap} is then averaged spatially and over time to obtain an estimate. The torque due to the capillary flow τ_{cap} is then estimated by considering the drag force experienced by the microplatelets when a fluid flows past it while assuming the microplatelets are fixed against the substrate so that they pivot about the point of contact with the substrate.

$$\tau_{cap} = \frac{1}{4} C_d \rho \pi b^3 {v_{cap}}^2 \quad (7)$$

where C_d is a geometric drag coefficient and b is the radius of the platelet.
The other relevant torques due to gravity τ_g and the magnetic force τ_m can be estimated by[3]:

$$\tau_g = 2\pi\Delta\rho a b^3 g cos\theta \quad (8)$$

$$\tau_m = \frac{2\pi\mu_0\chi_{ps}^2}{3(\chi_{ps}+1)}[(a+d)(b+d)^2 - ab^2]H_0^2(t)sin(2\theta) \quad (9)$$

$\Delta\rho$ is the difference in density between the platelet and the fluid, g is the gravitational constant, χ_{ps} is the magnetic susceptibility of the magnetic shell on the platelet, a is half of the platelet thickness, H_0 is the magnetic field intensity. By balancing all 3 torques present, the H_0 which corresponds to 0 net torque will be equivalent to B_{crit} at each value of v_{cap}.

Time interval between deposition of droplets during printing

To ensure good alignment in each voxel, the next droplet should be deposited on the existing structure before the underlying layer is fully dried. To make this process more systematic and reproducible, we recommend that a fresh droplet to be deposited on the existing structure based on a fixed time interval equivalent to 80% of the drying time of a droplet. The drying time of each droplet, t_{dry} should first be measured either by monitoring the side profile of the droplet using a microscope or by monitoring the mass of the droplet on a weighing balance if such a microscopy set up is unavailable. The overall drying time depends on the volume of each droplet and the environment of the user's laboratory (Supplementary Figure 12). The print steps can then be set to deposit one drop after a time interval, $t_{interval}$ = 0.8 t_{dry}. We found that this time interval ensures a consistent and continuous process. At shorter $t_{interval}$, there will be an eventual accumulation of solvent in the structure which will cause the fresh ink to spill out

from the existing structure and deteriorate the print resolution. At longer $t_{interval}$, the structure becomes close to being completely dried which may affect the alignment of the next droplet.

Multimaterial capacitor

A graphite-xirallic-graphite micropillar structure with diameter of approximately 3.0 mm was printed on a copper foil. The graphite layer heights were fixed at approximately 0.2 mm while the xirallic layer heights were varied between 0.25 mm to 0.65 mm. The as-printed structures were then infiltrated with an epoxy matrix. After curing, the top graphite layer was filed slightly to remove any excess of epoxy. The capacitance was measured using a multimeter (NT DT-9205A). To measure the discharging of the capacitors, larger capacitors with xirallic layer thickness of ~70 µm and total area ~ 5 cm^2 were printed to increase the total capacitance. A a resistance-capacitor (RC) circuit was built on a bread board using a 5-10 V power source and 1 MΩ resistors. The capacitors were charged for approximately 1 min to ensure complete charging before switching off the power source. The decrease in voltage across the capacitor was then monitored using the multimeter.

The capacitance of our printed device (~3 mm diameter, <1 mm high) was in the range of 0.1 nF. It is well established that the capacitance of parallel plate capacitors is determined by the area of the capacitor *A*, dielectric constant *ε* and height of the dielectric layer *d* through the relation:

$$C = \frac{\varepsilon A}{d} \tag{10}$$

The xirallic layer heights and alignments were then tuned to increase the resultant capacitance. As expected, the capacitance decreased with increasing xirallic layer height (Supplementary Figure 7a). Also, the devices with vertically aligned xirallic (θ = 90°) showed a consistently higher capacitance compared to devices with horizontally aligned xirallic (θ = 0°) layer. This is most likely due to the effects of crystallographic orientation on the resultant dielectric properties since the microplatelets were single crystals.[31] The capacitors were further tested by studying their discharging behaviour using a RC circuit to verify their functionality. Arrays of capacitors with xirallic thickness down to ~70 µm were printed to achieve a combined capacitance of up to ~120 nF for this measurement. Supplementary Figure 7b shows two sets

of discharging curves using different combinations of RC values. The discharging results matched the theoretical behaviour described by:

$$\frac{V}{V_0} = e^{-\frac{t}{RC}} \tag{11}$$

where *V* is the voltage across the capacitor, V_0 is the voltage across the charged capacitor, *R* is the resistance of the circuit and *C* is the capacitance of the capacitor.

Empirical model for pressure sensor

The model used to predict the sensor output of mixed alignment graphite sensor was based off the empirical data obtained from the stress-strain curve measurements (Fig. 6d) and the individual piezoresistive response from the θ = 0° and θ = 90° samples. Mathematically, two relationships were obtained for each alignment:

$$\sigma_\theta = f_\theta(\varepsilon_\theta) \tag{12}$$

$$\kappa_\theta = g_\theta(\phi) \tag{13}$$

where σ is stress, ε is strain, κ is electrical conductivity, ϕ is the graphite volume fraction. The subscripts represent the microplatelet alignment. The volume fraction of graphite in the composite is independent on orientation, therefore there is no subscript in ϕ here. In addition, the strain experienced by the material and the resultant volume fraction of the fillers within the material is related by the following equation:

$$\frac{\phi}{\phi_0} = \frac{1}{(1-\varepsilon_\theta)(1-v\varepsilon_\theta)^2} \tag{14}$$

Here, ϕ_0 refers to the volume fraction of graphite in the unstrained composite material and *v* is the Poisson's ratio of the material, taken to be ~ 0.5 for PDMS based materials.

When the sensor is compressed under an applied stress of σ, both θ = 0° and θ = 90° layers experience the same σ since they are stacked onto each other. Using equation (12), ε_0 *and* ε_{90} can be estimated. The strain can be converted into ϕ_θ using equation (14). κ_θ can then be estimated using equation (13) and the resistance in each layer can be obtained by factoring in the dimensions of the micropillar at each applied stress. This can then be converted into the final output signal obtained.

Supplementary Figures

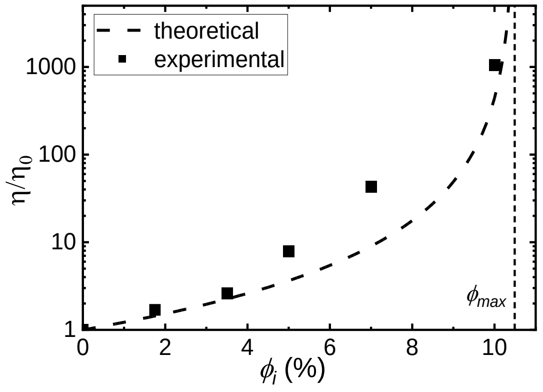

Supplementary Figure 1: Rheology of xirallic ink. Relation of the relative viscosity of xirallic inks, η/η_0 with varying initial microplatelet concentration, ϕ_i. The experimental values are based on the Krieger–Dougherty relation. ϕ_{max} corresponds to the maximum xirallic content the ink can hold.

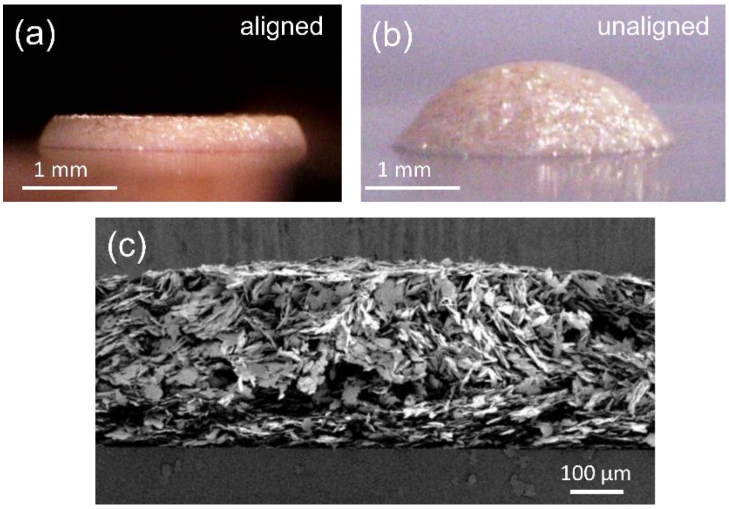

Supplementary Figure 2: Effect of magnetic field on droplet shape and microstructure. Optical images of the profile of a xirallic droplet (a) aligned with magnetic field and (b) dried without magnetic field. (c) SEM cross section of the microstructure of a xirallic droplet dried without magnetic field.

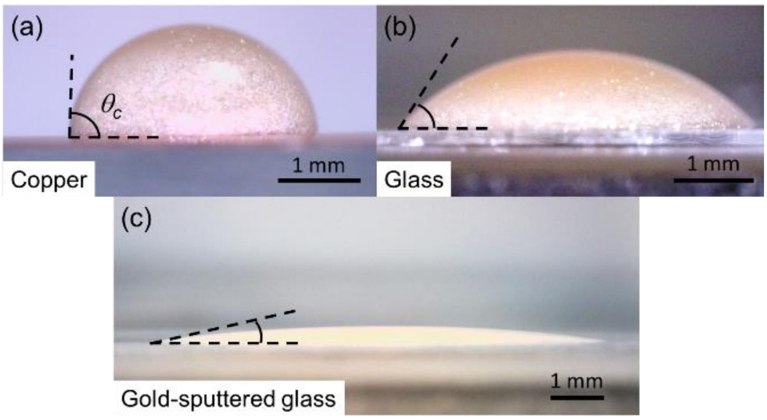

Supplementary Figure 3: Effect of substrate on droplet contact angle. Optical images of xirallic ink droplets deposited on different substrates: (a) copper, (b) glass and (c) gold-sputtered glass. The angles marked are the contact angles, θ_c of each droplet.

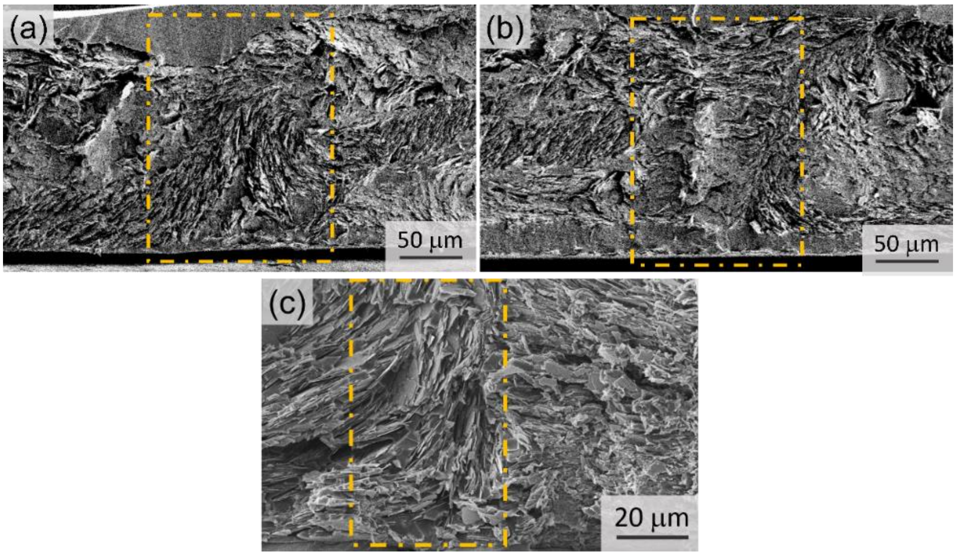

Supplementary Figure 4: Boundary between horizontally adjacent voxels. (a) and (b) High magnification SEM images of the boundary between voxels in the sample presented in Figure 4c in the manuscript. The adjacent voxels have different microplatelet orientations. (c) Boundary between adjacent voxels with the same microplatelet alignments. The areas marked out by the yellow box highlights the misaligned region in each sample.

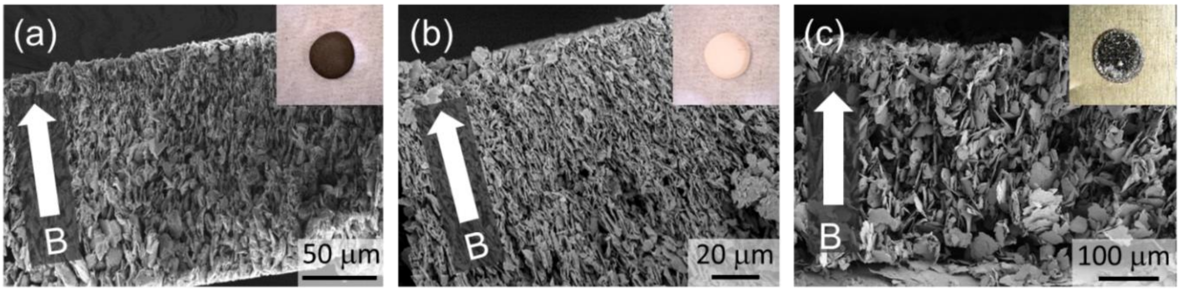

Supplementary Figure 5: Droplet printing of different material inks. SEM images of aligned droplets with different materials: (a) graphite, (b) hBN and (c) copper. The insets show the optical image of the corresponding printed droplets.

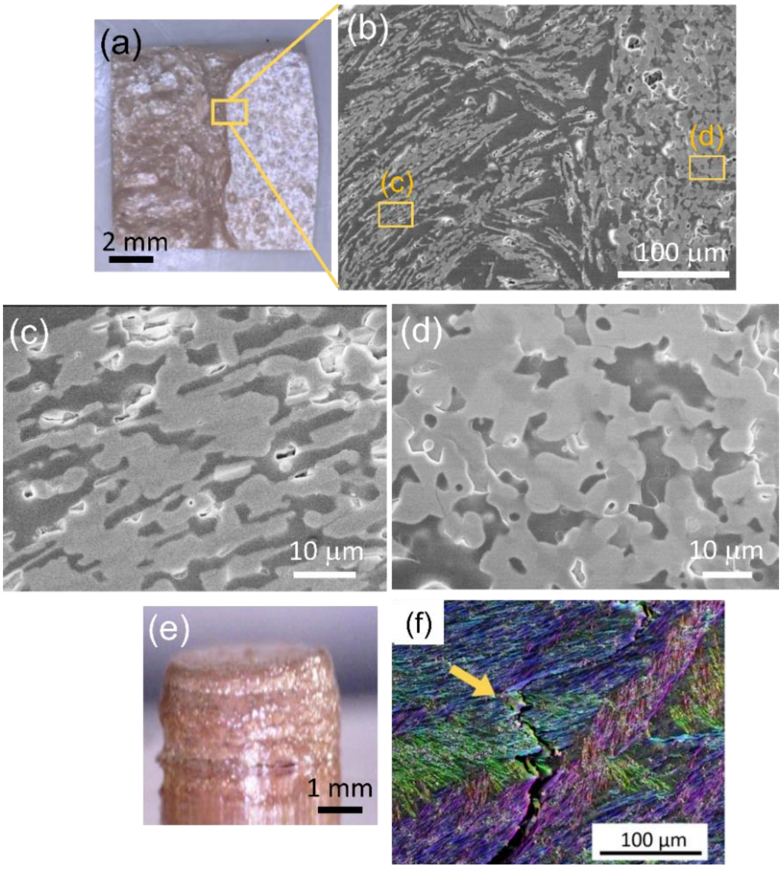

Supplementary Figure 6: Sample fabricated for mechanical testing. (a) Optical image of MDOD printed sintered xirallic sample used for mechanical testing. The different shades correspond to different microplatelet orientation. The lighter portion corresponds to θ = 0° (horizontally aligned) and the darker portion corresponds to θ = 90° (vertically aligned). (b) Microstructure of the sample at the boundary to show the different microplatelet orientations. (c) and (d) High magnification SEM images of the microstructure in each region, showing that the microstructure is as expected. (e) Optical image of xirallic-PDMS composite for compression test. (f) Electron micrograph showing a crack deflection event in a multilayer xirallic-epoxy composite with varying alignments. The colour code corresponds to microplatelets with varying alignment angles.

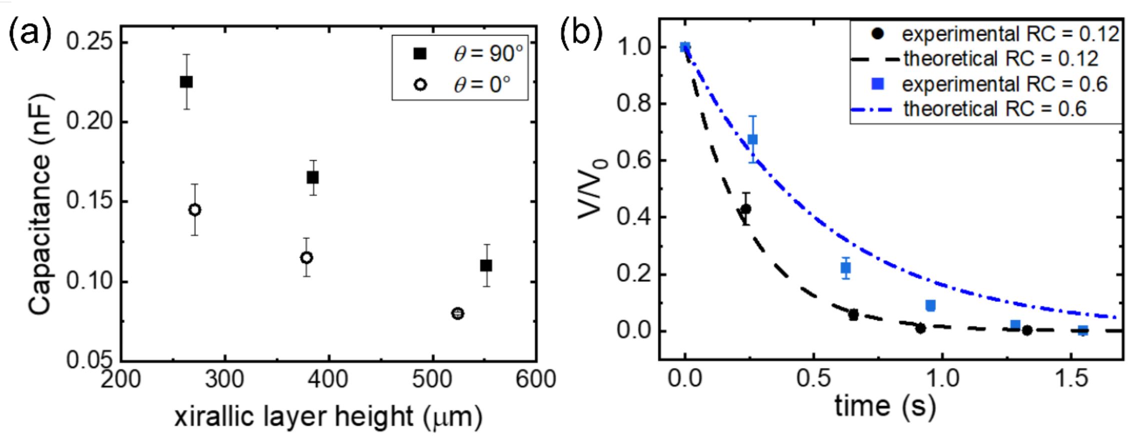

Supplementary Figure 7: Performance of MDOD printed capacitors. (a) Variation of the measured capacitance with the xirallic dielectric layer height for xirallic platelet orientations θ of 0° and 90°. (b) Capacitor discharge characteristics in a simple circuit with time constant RC values of 0.12 (black) and 0.6 (blue) as indicated in the legend. All error bars represent the standard deviation of the measurements.

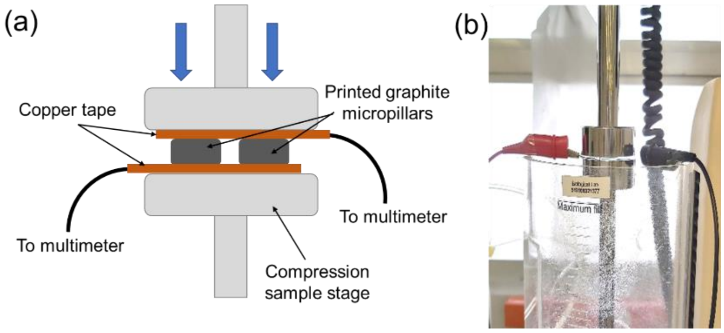

Supplementary Figure 8: Setup for printed piezoresistive graphite micropillar sensor. (a) Schematic of the testing setup to characterize the electrical response of the sensor material to applied compressive pressure. (b) Photograph of the actual setup used.

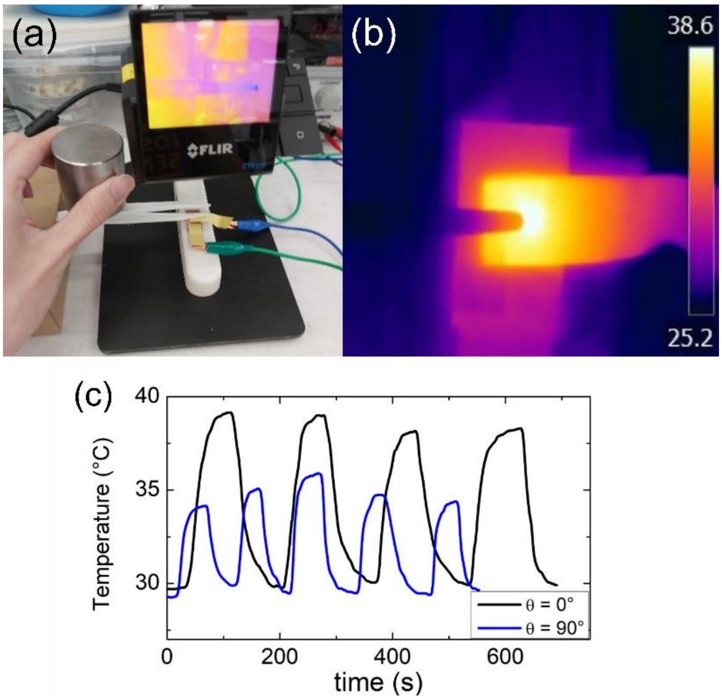

Supplementary Figure 9: Setup for thermal characterization of graphite sensor. (a) Setup used to study the Joule heating effect when pressure is applied onto the sensor. (b) thermal camera image showing the increase in temperature when a pressure of 25 kPa is applied onto the sensor. (c) Temperature of sensor under several cycles of loading and unloading of 25 kPa load for hBN alignments of 0° (black) and 90° (blue).

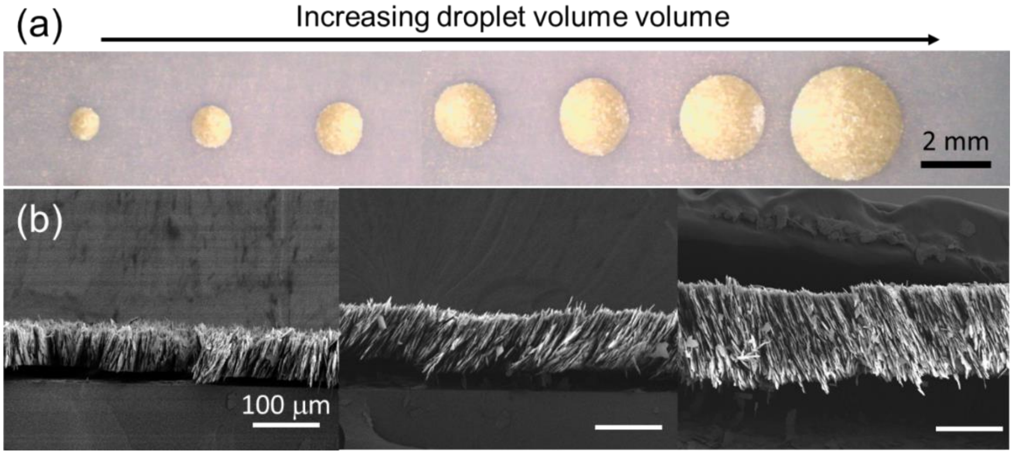

Supplementary Figure 10: Control of printing resolution. (a) Top view optical and (b) cross-sectional electron micrographs of printed xirallic droplets with varying droplet volumes. The smallest droplet printed is approximately 0.7 mm in diameter and the lowest vertical layer thickness achievable is approximately 50 mm.

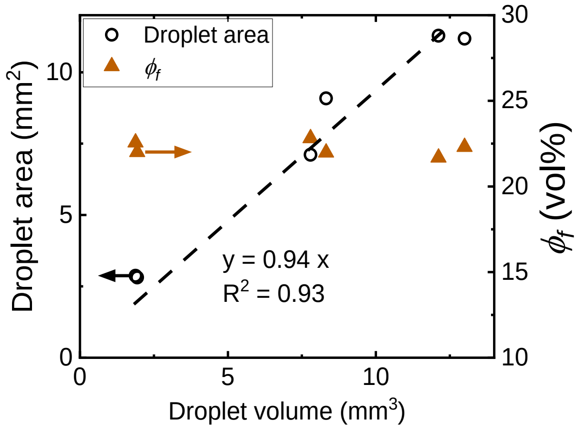

Supplementary Figure 11: Effect of droplet volume on platelet concentration. Variation of droplet area and final microplatelet concentration with varying droplet volume. The droplet area varies directly with droplet volume, leading to similar degree of densification during drying. Brown data points represent ϕ_f of the droplets and black data points represent the droplet areas.

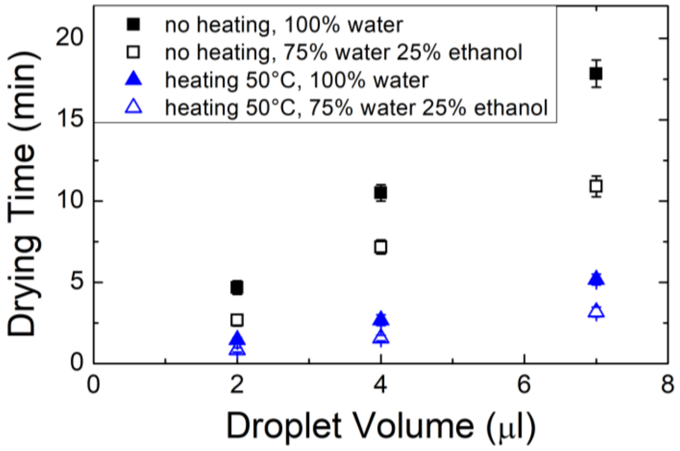

Supplementary Figure 12: Droplet drying time. Time taken for droplets to dry under ambient conditions, with additional substrate heating at 50 °C, and with ethanol as an ink cosolvent. Blue data points represent droplets drying under heating, while black data points represent droplets drying without heating. All error bars represent the standard deviation of the drying time.

Supplementary Table

Supplementary Table 1: Optimised ink composition for different microplatelet inks

Material	ϕ_i (vol%)	PVP content (wt%)
Xirallic	5-7	1
hBN	10-15	1
Graphite	10-15	1
Copper	5	2

Supplementary References

1. Deegan, R. D. *et al.* Contact line deposits in an evaporating drop. *Phys. Rev. E* **62**, 756–765 (2000).
2. Hu, H. & Larson, R. G. Evaporation of a sessile droplet on a substrate. *J. Phys. Chem. B* **106**, 1334–1344 (2002).
3. Erb, R. M., Segmehl, J., Schaffner, M. & Studart, A. R. Temporal response of magnetically labeled platelets under dynamic magnetic fields. *Soft Matter* **9**, 498–505 (2013).
