# Supplementary material for: Magnetically assisted drop-on-demand 3D printing of microstructured multimaterial composites
Source: Nat Commun. 2022 Aug 26;13:5015. doi: 10.1038/s41467-022-32792-1 (PMC9418172; doi:10.1038/s41467-022-32792-1)
Supplement: Supplementary file 3 — Description of Additional Supplementary Files [file 41467_2022_32792_MOESM3_ESM.pdf]

Description of Additional Supplementary Files

File Name: Supplementary Movie 1
Description: Video showing flow of solvent from fresh droplet into the existing droplet during printing.

File Name: Supplementary Movie 2
Description: Video showing the pressure sensor signal when a coin is placed onto the sensor area.

File Name: Supplementary Movie 3
Description: Video showing one instance of the electromechanical characterisation of the pressure sensor.

File Name: Supplementary Movie 4
Description: Video showing the printing process during magnetically assisted drop-on-demand printing.
